# Parthenogenic Blastocysts Derived from Cumulus-Free *In Vitro* Matured Human Oocytes

**DOI:** 10.1371/journal.pone.0010979

**Published:** 2010-06-07

**Authors:** Sohyun L. McElroy, James A. Byrne, Shawn L. Chavez, Barry Behr, Aaron J. Hsueh, Lynn M. Westphal, Renee A. Reijo Pera

**Affiliations:** 1 Center for Human Embryonic Stem Cell Research and Education, Institute for Stem Cell Biology and Regenerative Medicine, Stanford University, Palo Alto, California, United States of America; 2 Department of Obstetrics and Gynecology, Stanford University, Palo Alto, California, United States of America; 3 Division of Reproductive Endocrinology and Infertility, Stanford Hospital and Clinics, Palo Alto, California, United States of America; Katholieke Universiteit Leuven, Belgium

## Abstract

**Background:**

Approximately 20% of oocytes are classified as immature and discarded following intracytoplasmic sperm injection (ICSI) procedures. These oocytes are obtained from gonadotropin-stimulated patients, and are routinely removed from the cumulus cells which normally would mature the oocytes. Given the ready access to these human oocytes, they represent a potential resource for both clinical and basic science application. However culture conditions for the maturation of cumulus-free oocytes have not been optimized. We aimed to improve maturation conditions for cumulus-free oocytes via culture with ovarian paracrine/autocrine factors identified by single cell analysis.

**Methodology/Principal Finding:**

Immature human oocytes were matured *in vitro* via supplementation with ovarian paracrine/autocrine factors that were selected based on expression of ligands in the cumulus cells and their corresponding receptors in oocytes. Matured oocytes were artificially activated to assess developmental competence. Gene expression profiles of parthenotes were compared to IVF/ICSI embryos at morula and blastocyst stages. Following incubation in medium supplemented with ovarian factors (BDNF, IGF-I, estradiol, GDNF, FGF2 and leptin), a greater percentage of oocytes demonstrated nuclear maturation and subsequently, underwent parthenogenesis relative to control. Similarly, cytoplasmic maturation was also improved as indicated by development to blastocyst stage. Parthenogenic blastocysts exhibited mRNA expression profiles similar to those of blastocysts obtained after IVF/ICSI with the exception for *MKLP2* and *PEG1*.

**Conclusions/Significance:**

Human cumulus-free oocytes from hormone-stimulated cycles are capable of developing to blastocysts when cultured with ovarian factor supplementation. Our improved IVM culture conditions may be used for obtaining mature oocytes for clinical purposes and/or for derivation of embryonic stem cells following parthenogenesis or nuclear transfer.

## Introduction

Approximately 20% of oocytes routinely retrieved following hormone stimulation are classified as immature (termed germinal vesicle (GV) or metaphase I (MI) stage); these oocytes are discarded due to their reduced potential for embryo development under current culture conditions [1]. However, this cohort of oocytes is useful for studies aimed at elucidating the mechanisms of *in vitro* maturation of human oocytes and might ultimately contribute to the pool of embryos available for embryo transfer [2]. In addition, cumulus-free human oocytes may provide a platform for derivation of patient-specific embryonic stem cells (hESCs) including parthenogenic embryonic stem cells (pESCs) or somatic cell nuclear transfer-embryonic stem cells (SCNT-ESCs). Since the first report of *in vitro* human oocyte maturation in 1969 [3], several reports have documented blastocyst (BL) development or live birth achieved from oocytes matured *in vitro* [1], [4], [5]. However, nuclear and cytoplasmic maturation of cumulus-free oocytes remains suboptimal at best [2], [6], [7]. Moreover, ability of this cohort of oocytes to support the development of parthenogenic or nuclear transfer embryos to blastocyst stage has not been assessed.

The cumulus cells that surround oocytes within follicles modulate nuclear and cytoplasmic maturation by both physical cell-cell contact and the combined actions of paracrine factors [8]. However, during the process of intracytoplasmic sperm injection (ICSI), cumulus cells must be removed from oocytes. When these cumulus-free oocytes are directly exposed to commercially-available maturation media, they subsequently exhibit delayed oocyte maturation and abnormal embryo development [1], [2]. In the majority of studies of *in vitro* maturation (IVM), follicle stimulating hormone (FSH), luteinizing hormone (LH), estradiol and epidermal growth factor (EGF) are used as supplements. However, whether human oocytes express the receptors for FSH and LH is controversial [9], , and it is known that EGF affects oocyte maturation through the EGF receptor (EGFR), which is expressed by cumulus cells rather than the oocyte [11]. As outlined below, we used a microfluidic quantitative PCR (qPCR) system to elucidate the gene expression profiles of individual human oocytes and small numbers of cumulus cells using a combination of a large number of samples and targets [12], and then extended our studies via the use of parthenogenesis, in conjunction with gene expression profiling, as a functional assay of cytoplasmic maturation of oocytes.

Parthenogenesis *in vitro* is accomplished by artificial activation without sperm contribution [2]. *In vivo*, mammalian parthenotes are not able to develop to term due to aberrant, uniparental imprinting and consequent developmental abnormalities [13], [14]. Parthenogenesis, which can be induced *in vitro* by various electric, mechanical or chemical stimulations, is a valuable functional assay of oocyte developmental competence [15]. Also, pESC lines, that carry isogenic genome information from the oocyte donors, can be derived and may represent a potential alternative source of stem cells for basic scientific studies as well as novel therapeutic application. Although human pESCs have been derived from different oocyte sources such as donated mature or cryopreserved oocytes [16], [17], *in vitro*-matured oocytes present a potentially-abundant source for production of parthenogenic blastocysts.

Here, we report improved methods for *in vitro* maturation of cumulus-free human oocytes by supplemented culture media with ovarian paracrine/autocrine growth factors that were selected on the basis of gene expression profiles of single human oocytes and associated cumulus cells. Then we tested functionality of oocytes matured *in vitro* by assessing the ability to develop to the blastocyst stage, and by comparing the gene expression profiles of parthenogenic embryos and those obtained from IVF/ICSI.

## Results

### Expression of genes for paracrine/autocrine ligands and their cognate receptors in oocytes and cumulus cells

**Figure 1:**
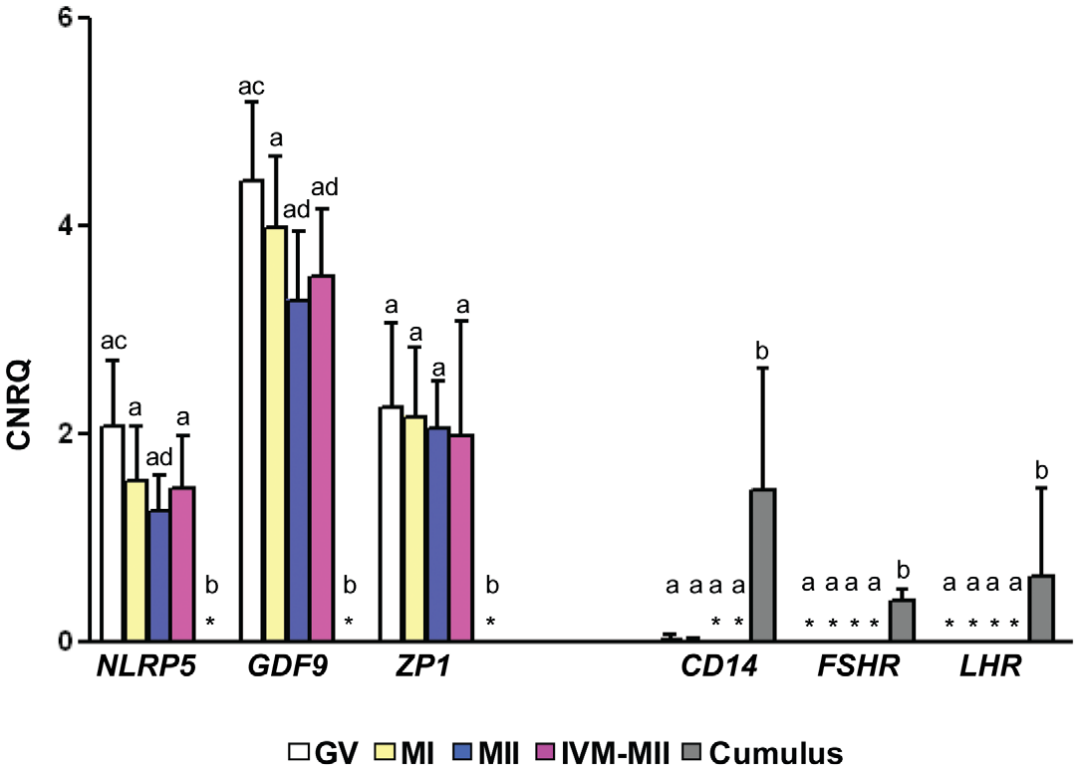
Expressions of oocyte-specific or cumulus cell-specific genes. Values are the means of different stages of oocytes including GV (N = 10), MI (N = 8), MII (N = 9), IVM-MII (N = 10) and cumulus cells (N = 5). Error bars represent standard deviation (SD). CNRQ: calculated normalized relative quantity. *: Not detected in all samples. ^a,b^ or ^c,d^: Values were significantly different (P<0.05).

In order to improve culture conditions for maturation of cumulus-free oocytes *in vitro*, we began by assaying the expression of 15 growth factors (BDNF, IGF-I, estradiol, GDNF, leptin, FGF1, FGF2, GM-CSF, EGF, TGF-α, TGF-β1;2;3, and ET-1;2) and 27 of their cognate receptors that have been shown to regulate oocyte maturation. Selection was based on published literature deposited in two online databases (http://ovary.stanford.edu/ and http://receptome.stanford.edu/HPMR/) [18], [19]. Four different stages of oocytes, striped of their zona pellucida (ZP), were assayed: 1) GV (germinal vesicle), 2) MI (metaphase I), 3) MII (spontaneously matured metaphase II before IVM) and 4) IVM-MII (*in vitro* matured metaphase II). We confirmed the purity of ZP-free oocytes and cumulus cell samples based on the exclusive expression of known oocyte-specific and cumulus cell-specific genes. As expected, expression of the genes *NLRP5*, *GDF9* and *ZP1* was limited to oocytes, while expression of the genes *CD14, FSHR* and *LHR*, was limited to cumulus cells (Figure 1).

**Figure 2:**
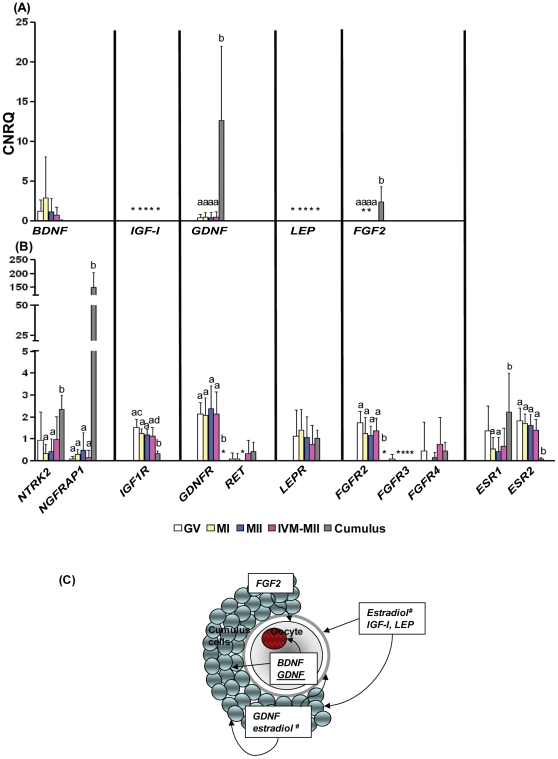
Expressions of ovarian paracrine/autocrine factors and their cognate receptors selected for supplementation in *in vitro* maturation media. (A) Ovarian paracrine/autocrine ligands, (B) their cognate receptors, and (C) schematic representation of gene expressions. Values are the means of different stages of oocytes including GV (N = 10), MI (N = 8), MII (N = 9), IVM-MII (N = 10) and cumulus cells (N = 5). Error bars represent standard deviation (SD). CNRQ = calculated normalized relative quantity. *: No detected in all samples. ^a,b^ or ^c,d^: Values were significantly different (P<0.05), underline: low expression level, #: adopted from Hillier et al. [46].

We examined expression of a set of candidate receptors and ligands in oocytes and cumulus cells, respectively (Figure 2A, 2B and S1). Among the growth factors, expression of mRNA of genes that encode for GDNF, FGF2, GM-CSF and TGF-β1 was significantly higher in cumulus cells than in oocytes. Conversely, the majority of receptors were detected at the mRNA level in oocytes with the exception for *FGFR3*, *CSF2Rβ*, *EGFR* and *TGF-βR2*. Among the receptors, expression of mRNA of genes that encode for NTRK2, NGFRAP1, ESR1 and EDNRA was higher in cumulus cells, while oocytes had significantly higher expression of mRNA of genes that encode for IGF1R, ESR2 and TGF-βR3. There were a few genes that were detected exclusively in only cumulus cells (CSF2Rβ and EGFR) or oocytes (GDNFR, FGF2R). We observed that even though the expression of the ligand, *BDNF* was low in cumulus cells compared to oocytes, its receptors, NTRK2 and NGFRAP1 were expressed in both oocytes and cumulus cells. In particular, NGFRAP1 was expressed more than 350 fold higher in cumulus cells than in oocytes. We also noted that mRNA of only two genes (*IGF-I* and *leptin*) was not expressed at detectable levels in either compartment, oocytes or cumulus cells. However, mRNA corresponding to their receptors, *IGF1R* and *LEPR*, was expressed in both oocytes and cumulus cells.

Six ovarian factors (BDNF, IGF-I, estradiol, GDNF, leptin and FGF2) were further studied based upon the expression of their receptors in oocytes and cumulus cells described above, as well as the availability of growth factor concentration information from previous animal model studies [1], [20], [21], [22], [23]. We derived a protocol for paracrine/autocrine supplementation for *in vitro* maturation of cumulus-free oocytes that includes supplementation with BDNF, IGF-I, GDNF, leptin, FGF2 and estradiol as shown in Figure 2C.

### Effect of ovarian paracrine/autocrine supplement on oocyte nuclear maturation

To test whether culture supplementation to promote nuclear maturation, we assessed the polar body extrusion after culture of immature GV and MI oocytes in three different maturation conditions: 1) Commercial human oocyte maturation medium (Sage), 2) IVM-medium, and 3) IVM-medium supplemented with selected ovarian factors as described above and in Materials and Methods. As shown in Table 1, as expected, nuclear maturation of GV oocytes was less efficient than MI oocytes. Among the GV oocytes, 50–68% of oocytes extruded a first polar body within 48 h in the three different media. Significant differences were not observed with the GV oocyte nuclear maturation between any of the three media. In contrast, nuclear maturation of MI oocytes was much more efficient with values ranging from 75 to 94% in the three different media. The supplementation of MI oocytes in IVM-medium with ovarian factors demonstrated significantly higher rates of extruding a first polar body (94%) relative to media without supplementation (75%) (Table 1).

**Table 1:** Nuclear maturation of cumulus-free human oocytes *in vitro* from hormone-stimulated cycle.

Oocytes	Culture media	No. of oocytes	Nuclear maturation rate (%)	
			24 h	48 h
GV	Sage	45	22 (48.9)	31 (68.9)
	IVM-medium	46	19 (41.3)	23 (50.0)
	Supplement	98	45 (45.9)	53 (54.1)
MI	Sage	42	37 (88.1)	37 (88.1)
	IVM-medium	29	21 (72.4)^a^	22 (75.9)^a^
	Supplement	51	48 (94.1)^b^	48 (94.1)^b^

Sage: commercial IVM medium supplemented with 10% SPS, FSH, hCG and estradiol.

IVM-medium: IVM-medium supplemented with 10% SPS.

Supplement: IVM-medium supplemented with 10% SPS, BDNF, estradiol, IGF-I, GDNF, FGF2, leptin.

GV: germinal vesicle stage.

MI: metaphase I stage.

Nuclear maturation: the presence of a first polar body.

a,b Within columns, values with different superscripts were significantly different (P<0.05).

### Functional analysis of ovarian paracrine/autocrine supplement via assessment of parthenote development

We used parthenogenic development as a functional assay of cytoplasmic maturation. Following chemical activation of oocytes matured *in vitro*, we monitored cleavage and development of the parthenotes daily. We observed that following activation, culture in both Sage and IVM-supplementation media demonstrated significantly higher cleavage rates (87.2% and 82.3%, respectively) compared to IVM-medium alone (55.6%). Most notably, however, parthenogenic blastocysts were only produced from embryos cultured with supplementation and not with Sage or IVM-medium alone (Table 2 and Figure S2).

**Table 2:** Embryo development after parthenogenetic activation of human oocytes matured *in vitro*.

IVM media	Total oocytes	Activated (%)*	2-cell (%)*	8-cell (%)**	Blastocysts (%)**
Sage	66	47 (71.1)	41 (87.2)^b^	4 (9.8)	-
IVM-medium	44	36 (81.8)	20 (55.6)^a^	3 (15.0)	-
Supplement	77	62 (80.5)	51 (82.3)^b^	7 (13.7)	3 (5.9)

Sage: commercial IVM medium supplemented with FSH, hCG and estradiol.

IVM-medium: IVM-medium supplemented with 10% SPS.

Supplement: IVM-medium supplemented with BDNF, estradiol, IGF-I, GDNF, FGF2, leptin.

*Percentage from activated oocytes.

**Percentage from cleaved embryos.

a,b Within columns, values with different superscripts were significantly different (P<0.05).

### Gene expression profiling of human parthenotes

**Figure 3:**
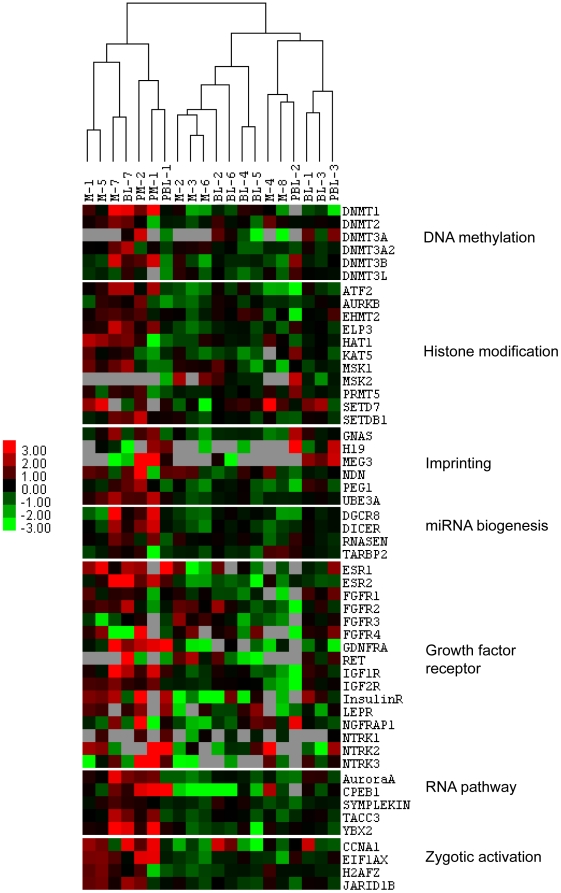
Treeview display of cluster analysis of gene expression of all morulas and blastocysts from parthenogenesis and IVF/ICSI. PM = parthenogenic morula, PBL = parthenogenic blastocyst.

**Figure 4:**
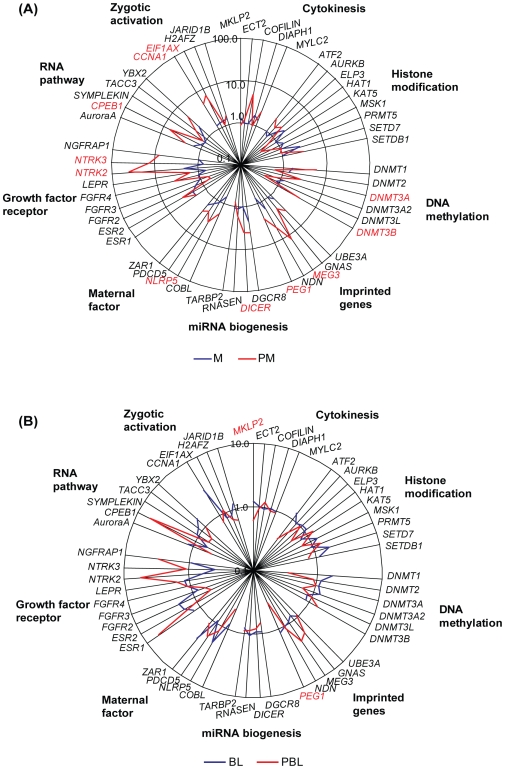
Gene expression of parthenogenic embryos. The average mRNA expression level of morulas (A) and blastocysts (B) from parthenogenesis and IVF/ICSI were displayed. Red color: Significantly differentially expressed. PM = parthenogenic morula, PBL = parthenogenic blastocyst.

A total of 67 genes, from 10 functional categories related to embryo development were selected for analysis of parthenogenic and IVF/ICSI embryos. The categories included RNA pathway (5 genes), zygotic activation (4 genes), histone modification (10 genes), DNA methylation (6 genes), imprinting (6 genes), miRNA biogenesis (4 genes), pluripotency (3 genes), growth factor receptors (20 genes), maternal factors (4 genes) and cytokinesis (5 genes). These genes were selected for analysis of parthenogenic and IVF/ICSI embryos at the morula and blastocyst stage based primarily on published reports from non-human model systems (Figure 3, 4A, 4B and Table S2). In our data analysis, we observed 97% similarity in mRNA expression between parthenotes and IVF/ICSI embryos at the blastocyst stage (Figure 3 and 4B), with reduced similarity at the morula stage (83.6%, Figure 3 and 4A). More specifically, only the mRNAs of genes that encode for MKLP2 and PEG1 were expressed at lower levels in parthenogenic blastocysts in comparison to IVF/ICSI blastocysts (Figure 4B). Genes that encode for DNMT3A and MEG3 were expressed only in parthenotes but not in IVF/ICSI at the morula stage. Parthenogenic morula also had significantly higher expression levels of the mRNAs for DNMT3B, PEG1, DICER, NLRP5, NTRK2, NTRK3, CPEB1, CCNA1 and EIF1AX (Figure 4A).

## Discussion

In this study, we first used a microfluidic quantitative PCR system to elucidate the gene expression profiles of single human oocytes and small groups of cumulus cells and subsequently improved upon *in vitro* maturation of cumulus-free oocytes to obtain parthenogenic blastocysts. We also observed that parthenotes from *in vitro* matured oocytes have similar mRNA expression levels of the majority of studied genes compared to IVF/ICSI embryos at the blastocyst stage.

Analysis of gene expression in single human oocytes and small groups of cumulus cells revealed notable similarities and differences from previous reports including human and model organisms. FSH, LH, estradiol and EGF are commonly used to promote maturation of cumulus-enclosed oocytes. According to the mRNA expression analysis in our study, there is negligible expression of the receptors for FSH, LH and EGF in human oocytes. This is consistent with a recent animal study [9] but contradictory to a previous report showing *FSHR* and *LHR* mRNA expression in mouse oocytes and pre-implantation embryos [10]. Furthermore, parthenote development in Sage medium with hormone supplementation did not improve late stage (further than 8-cells) embryo development compared to IVM-medium alone. Our gene expression and embryo development data suggest that FSH and LH may not affect oocyte nuclear and cytoplasmic maturation directly. In contrast, both *ESR1* and *ESR2*, the receptors for estradiol, were detected in oocytes as well as cumulus cells in our study, suggesting a role for estradiol in oocyte maturation [24]. Therefore, the higher cleavage rate in Sage medium compared to IVM-medium alone is most likely due to the estradiol supplementation in Sage medium.

Both animal and human studies have demonstrated the important roles of neurotrophins (BDNF, NTF3 and NTF4) in oocyte maturation mediated by their receptors (NTRK1–3 and NGFRAP1) [20], [25]. We observed expression of *BDNF* mRNA and its receptors (*NTRK2* and *NGFRAP1*) in both oocytes and cumulus cells. The higher expression of *NGFRAP1* in cumulus cells suggests that BDNF may have a role in cumulus cell functions. It is important to note that the probe for *NTRK2* in our study detected both the full length as well as the truncated forms of *NTRK2*. We suspect that the *NTRK2* mRNA expression observed in the present study is the truncated form, since the full length form was not detected in either human oocytes or cumulus cells previously [26]. Interestingly, *NGFRAP1* was detected in oocytes in our study but not in the previous study [26]. This may reflect the greater sensitivity of our microfluidic qPCR system over other traditional qPCR systems. Also, the previous study was conducted with cumulus-oocyte-complexes from small antral follicles without hormone stimulation, which may dramatically change the mRNA expression profiles compared to those undergoing hormone stimulation.

Expression of the mRNA encode for Glial cell line-Derived Neurotrophic Factor (GDNF) was detected in cumulus cells as shown previously [22] as well as oocytes in our study. GDNF is secreted by cumulus, granulosa, and theca cells as an ovarian factor stimulated by the preovulatory LH surge [22], and induces oocyte maturation and embryo developmental competence [27] by GDNFR and rearranged during transformation (RET). Based on the findings that GDNFR is only expressed in oocytes, and a low level of RET expression was detected in both oocytes and cumulus cells, our data suggest that GDNF might increase oocyte maturation by both a paracrine and autocrine manner in humans.

At the mRNA level, *FGF2* was highly expressed in cumulus cells, while its receptor *FGF2R* was highly expressed in oocytes in our study. FGF2 and its receptors have been shown to be expressed at the protein level by both oocytes and granulosa cells up to secondary follicles [28], but the expression patterns in antral follicles have not been studied to date. Based on our gene expression data, FGF2 could affect oocyte maturation in a paracrine manner via the cumulus cells. This notion is supported by a previous study showing a higher concentration of FGF2 in follicular fluid compared to serum [29] and suggests that FGF2 production from the ovary has pivotal functions during follicle development and oocyte maturation.

Two parthenogenic blastocysts were produced from *in vitro* matured GV oocytes from hormone-stimulated cycles in our study. This is a proof of principle to demonstrate that immature oocytes are able to achieve the developmental competence with selected ovarian factors *in vitro*. In spite of ovarian factor supplementation, nuclear maturation of GV oocytes was not improved compared to MI stage oocytes. It may be due to the fact that the majority GV oocytes might be collected from pre-antral follicles which are not able to resume meiosis [30]. However, detailed protein expression of individual ovarian factors and their receptors in oocytes need to be further studied in order to elucidate the factors that may affect oocytes in specific maturation stages.

Once we had optimized the *in vitro* maturation of oocytes, we were able to obtain parthenogenic morulas and blastocysts in culture. It is noteworthy that this is the first study to show the gene expression profiles of human parthenogenic embryos at any stage. Overall, parthenotes have similar mRNA expression levels as IVF/ICSI embryos at the blastocyst stages (Figure 3 and 4B). The altered expression patterns of Cyclin A1 (CCNA1) in parthenotes changed from high (in morula) to low (in BLs) compared to IVF/ICSI embryos, which may due to it inefficient zygote genomic activation [31]. The altered expression of CCNA1 is similar to the expression of EIF1AX, another marker for zygotic genomic activation in our study as well as in a previous animal study [32]. However, we cannot suspect any abnormal effect on parthenotes due to the aberrant expression of CCNA1 since the protein is not expressed at the blastocyst stage [33], and its cell cycle regulation role is limited in meiotic cells [34]. Higher expression of cytoplasmic polyadenylation element-binding protein 1 (*CPEB1*) in parthenotes compared to normal embryos may increase cell cycle arrest and cellular senescence by inducing p53 translation [35]. This may cause lower cell number and higher apoptosis in parthenotes compared to normal embryos [14].

The mRNA expression pattern of growth factor receptors in parthenotes were different compared to normal embryos, suggesting that growth factors supplemented in oocyte/embryo culture media may alter the expression of their receptors [36]. Interestingly, BDNF improves pregnancy rates by increasing trophoblast cell growth and survival via its receptor NTRK2 expressed by the trophectoderm of blastocysts [37]. The effect of higher expression of *NTRK2* and *NTRK3* mRNA in parthenotes need to be further studied.

Aberrant imprinting is the major cause of embryo lethality of parthenogenic embryos. In our study, morula stage parthenotes exhibited high expression levels of *MEG3* and *PEG1*. Maternally expressed MEG3 is regulated by a intergenic differentially methylated region in human gametes and embryos [38]. A possible explanation for this finding is that residual mRNA expression from oocytes was still present in morula stage parthenotes and/or chemical activation induced the aberrant expression of certain genes in parthenotes. Paternally expressed PEG1 exhibited significantly higher levels in parthenotes at the morula stage and dramatically decreased at the blastocyst stage. The high expression levels of two genes (*DNMT3A* and *DNMT3B*) related to DNA methylation in our study is also consistent with a mouse study showing the elevated methylation in parthenotes compared to IVF/ICSI embryos [39]. Analogous to CCNA1 and EIF1AX discussed above, the higher expression of de novo methyltransferase, *DNMT3B*, in parthenotes may be due to the altered zygotic activation since DNMT3B expression has been shown to be originated from embryo [40]. The regulation of epigenetic modifications in human pre-implantation embryos needs to be explored in further depth in order to understand the underlying molecular mechanisms behind early human development as well as the low efficiency of parthenogenesis and SCNT.

In our study, cleaved day 3 parthenotes were used to establish pESCs from single blastomeres using derivation methods as previously described [41]. Of the blastomeres isolated, approximately 21% (8 out of 38) divided once, but no blastocele could be detected in arrested parthenotes. Only blastomeres from non-arrested 8-cell stage parthenote formed blastoceles and proliferated (data not shown). From the 8 blastomeres isolated from an 8-cell parthenote, 3 blastomeres divided, which suggested that those 3 blastomeres are the most viable from the original 8-cell parthenote. This interpretation is supported by a previous study showing that the blastomeres from arrested human embryos still divided *in vitro* when isolated from the original embryos [42]. The results indicate the potential for individual blastomere growth and development from parthenotes obtained from *in vitro* maturation in spite of the fact that we did not derive a pESC line at this time. It would be useful to compare the potential of day 2 parthenotes which are more abundant since an ESC line was recently derived from 4-cell blastomeres [43].

In conclusion, cumulus-free human oocytes from hormone-stimulated cycles can be matured *in vitro* with the supplementation of ovarian paracrine/autocrine factors to achieve embryo developmental competence to blastocysts. Also, parthenogenic blatocysts from IVM oocytes exhibit similar gene expression profiles of selected genes compared to IVF/ICSI. Parthenogenesis is one of key steps towards successful human SCNT. Our improved IVM culture conditions may be used for supplying mature oocytes for regenerative medicine, including pESC and SCNT-ESC derivation.

## Materials and Methods

### Sample collection

Oocytes, cumulus cells and frozen embryos were collected from Stanford Fertility and Reproductive Medicine Center after approval by the Stanford Institutional Review Board (IRB); all samples were obtained with written informed consent from all participants involved in the study. Patients who had fewer than 50% mature oocytes were excluded from the oocyte donation. Protocols for controlled ovarian hyperstimulation and oocyte retrieval were as described previously [7]. Cumulus-free immature oocytes from consenting ICSI patients were obtained 6–8 h after oocyte retrieval and transferred to the research laboratory.

### Gene expression profiling of oocytes and cumulus cells

For analyses of gene expression in oocytes and cumulus cells, the following samples were collected; 10 GV (germinal vesicle), 8 MI (metaphase I), 9 MII (spontaneously matured metaphase II before IVM), 10 IVM-MII (*in vitro* matured metaphase II) oocytes, and 5 cumulus aggregates containing 5–10 cells of corona radiata. Oocytes and cumulus cells were collected from 21 patients undergoing ICSI treatment. Two GV oocytes were collected from 2 PCOS (polycystic ovarian syndrome) patients. Removal of the zona pellucid (ZP) was accomplished by treatment with acidic tyrode (Millipore Co., Billerica, MA), and samples were stored at −80°C until use. The BioMark Dynamic Array microfluidic qPCR system (Fluidigm Corporation, San Francisco, CA) was used for gene expressions analysis of oocytes and cumulus cells. Individual oocytes or small groups of cumulus cells were pre-amplified according to the manufacturer's protocol (Fluidigm Co.) using 20× Taqman gene expression assays (Applied Biosystems, Foster City, CA) as listed in Table S1. Reaction mix contained 2.5 µl 2× Universal Master Mix (Applied Biosystems), 0.25 µl Sample Loading Buffer (Fluidigm Co.), and 2.25 µl pre-amplified cDNA for loading into the sample inlets of the 48 by 48 Dynamic Array (DA) (Fluidigm Co.). For probes, the reaction mix contained 2.5 µl 20× Taqman gene expression assay and 2.5 µl Assay Loading Buffer (Fluidigm Co.) for loading into the assay inlets on the DA. Each sample was assayed in duplicate, and CNRQ (calculated normalized relative quantity) values were calculated by classic delta-delta-Ct method and normalized to the multiple housekeeping genes, large, P0 (*RPLPO*) and glyceraldehydes-3-phosphate dehydrogenase (*GAPDH*) as a control using the qBase*Plus* 1.3 analysis software (http://www.biogazelle.com) [44].

### *In vitro* maturation

Cumulus-free human oocytes from hormone stimulated ICSI patients were matured in three different conditions: 1) Sage (commercial oocyte maturation medium, Coopersurgical/Sage, Trumbull, CT) supplemented with 10% serum protein substitute (SPS), 0.075 IU/ml recombinant FSH (rFSH, Serono Laboratories, Randolph, MA), 0.025 µg/ml recombinant human chorionic gonadotrophin (rhCG, Serono Laboratories), 1 µg/ml estradiol (Sigma-Aldrich, St. Louis, MO) based on previous studies [1], [6], 2) IVM-medium based on previous report with minor modification of addition of 0.5 mg/l human transferrin supplemented with 10% SPS [1], [45], and 3) IVM-medium supplemented with 10% SPS and ovarian paracrine/autocrine factors [3 ng/ml of BDNF (PeptroTech Inc., Rocky Hill, NJ), 100 ng/ml of IGF-I (Sigma-Aldrich), 1 µg/ml of estradiol, 30 ng/ml of GDNF (R&D System Inc., Minneapolis, MN), 10 ng/ml of leptin (PeptroTech Inc.), and 0.5 ng/ml of FGF2 (R&D System Inc.)] based on results described above on expression of receptors in oocytes. A total of 189 GV and 122 MI cumulus-free oocytes from more than 90 patients were matured at 37°C in 6% CO_2_, 5% O_2_ and 89% N_2_. Nuclear maturation was characterized by the presence of a first polar body (metaphase II) at 24 and 48 h after IVM, and cytoplasmic maturation was determined by extent of early embryo development following chemical activation.

### Functional assessment of maturation via parthenogenesis and embryo culture *in vitro*

Following *in vitro* maturation, oocytes at metaphase II with the presence of a first polar body were activated using 10 µM calcium ionophore A23187 (Sigma-Aldrich) for 5 min followed by 2 mM DMAP (Sigma-Aldrich) for 4 h at 37°C in 6% CO_2_, 5% O_2_ and 89% N_2_. Activated oocytes were subsequently cultured in biphasic culture medium, Quinn's Cleavage and Blastocyst media (Coopersurgical) supplemented with 10% SPS and growth factors [10 ng/ml of BDNF, 40 ng/ml of IGF-I, 5 ng/ml of EGF (R&D System Inc.), 2 ng/ml of GM-CSF (R&D System Inc.), 0.5 ng/ml FGF2, and 10 ng/ml of GDNF] at 37°C in 6% CO_2_, 5% O_2_ and 89% N_2_. Embryo development was monitored via microscopy daily for 7 days.

### Gene expression profiling of parthenotes and IVF/ICSI embryos

For analyses of gene expression in embryos, cryopreserved day 3 embryos and day 6 blastocysts from 10 IVF/ICSI patients were thawed via an embryo thawing kit (Cooper Surgical) or a blastocyst thawing kit (Cooper Surgical), respectively, according to manufacturer's protocol. Eight morulas and seven blastocysts were harvested after culturing in Quinn's Blastocyst media supplemented with 10% SPS at 37°C in 6% CO_2_, 5% O_2_ and 89% N_2_. Two parthenogenic morulas and three parthenogenic blastocysts were also collected on day 5 and 6 of *in vitro* culture, respectively. The Taqman gene expression assays were as listed (Table S2). CNRQ values were calculated by classic delta-delta-Ct method and normalized to the multiple housekeeping genes, cadherin-associated protein, beta 1(*CTNNB1*) and *GAPDH* using the qBase*Plus* 1.3 analysis software. CNRQ values were converted into a log_2_ value, and imported into the clustering program, Cluster 3.0, and visualized by TreeView software (http://rana.lbl.gov).

### Statistical analysis

Oocyte maturation and embryo development data were entered into a two-by-two contingency table, and Fisher's exact test was used to generate P-values in Prism version 5.02 for windows (GraphPad Software, Inc.). Gene expression data (CNRQ) of oocytes and cumulus cells were analyzed by one-way ANOVA with Bonferroni post test in Prism version 5.02. Gene expression data of embryos were analyzed by one-way ANOVA in JMP® software (SAS Institute, Cary, NC). Significant differences between experimental groups were noted when the comparison-wise P-value was less than 0.05.

## Supporting Information

Figure S1 Expression data for ovarian paracrine/autocrine factors and their cognate receptors that were not subsequently used for supplementation in *in vitro*. (A) Paracrine/autocrine ligands, and (B) their cognate receptors. Values are the means of different stages of oocytes including GV (N = 10), MI (N = 8), MII (N = 9), IVM-MII (N = 10) and cumulus cells (N = 5). Error bars represent standard deviation (SD). CNRQ: calculated normalized relative quantity. *: Not detected in all samples. a,b or c,d: Values were significantly different (P<0.05).
(0.94 MB TIF)

Figure S2 Development of parthenogenic embryos. Embryos on day 1 (A) and day 3 (B,C); blastocysts on day 6 (D) and day 7 (E). Cells were stained with Hoechst 33342 (F). The images were taken either under 300× (A, B, E, F) or 200× (C, D) magnification.
(4.97 MB TIF)

Table S1 Taqman probe assay numbers for genes tested in oocytes and cumulus cells.
(0.06 MB DOC)

Table S2 Taqman probe assay numbers for genes tested in embryos.
(0.09 MB DOC)
