# Supplementary figures and images for: Parthenogenic Blastocysts Derived from Cumulus-Free In Vitro Matured Human Oocytes
Source: PLoS One. 2010 Jun 7;5(6):e10979. doi: 10.1371/journal.pone.0010979 (PMC2881862; doi:10.1371/journal.pone.0010979)

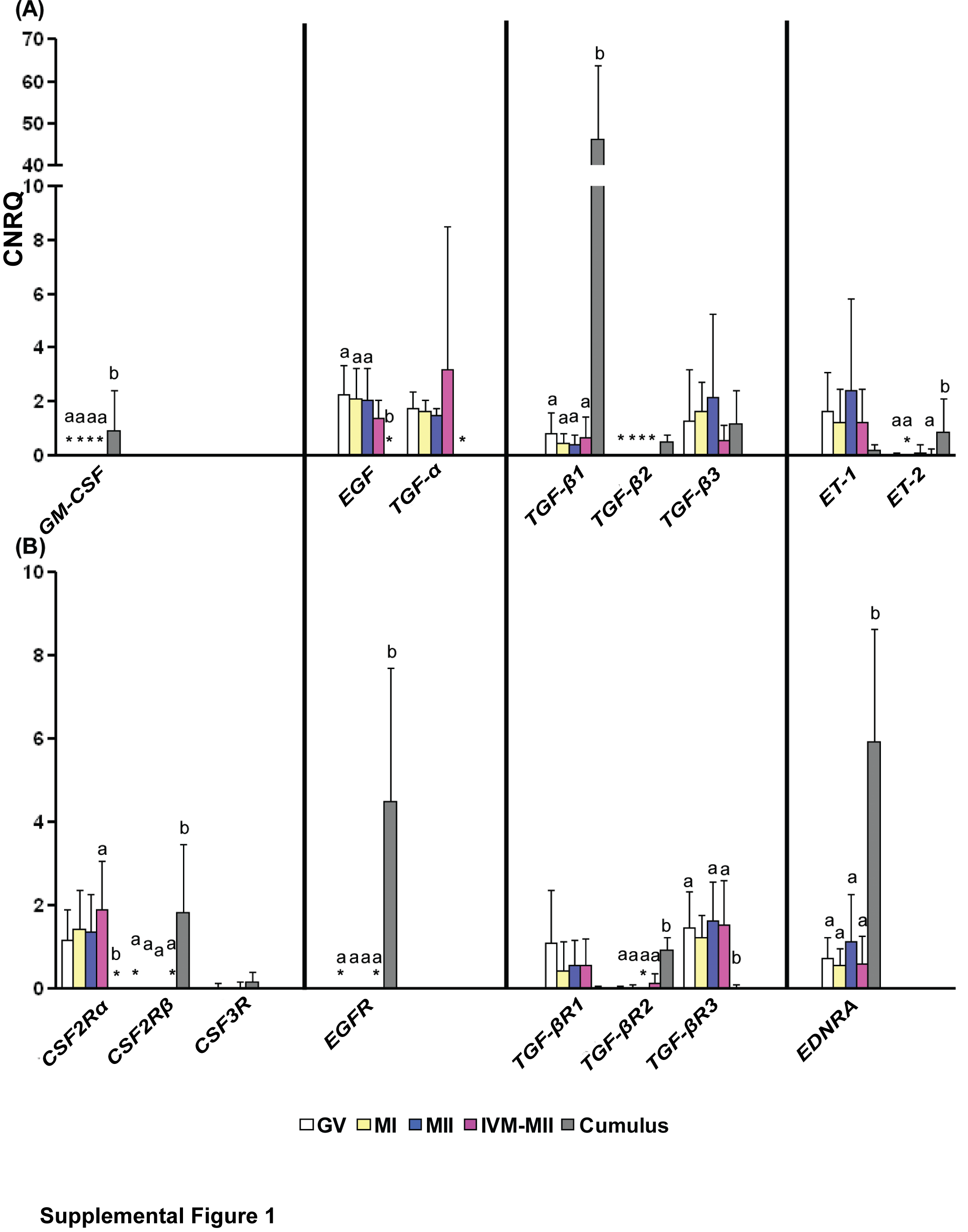

Supplement: Figure S1 — Expression data for ovarian paracrine/autocrine factors and their cognate receptors that were not subsequently used for supplementation in in vitro. (A) Paracrine/autocrine ligands, and (B) their cognate receptors. Values are the means of different stages of oocytes including GV (N = 10), MI (N = 8), MII (N = 9), IVM-MII (N = 10) and cumulus cells (N = 5). Error bars represent standard deviation (SD). CNRQ: calculated normalized relative quantity. *: Not detected in all samples. a,b or c,d: Values were significantly different (P<0.05). (0.94 MB TIF) [file pone.0010979.s001.tif]

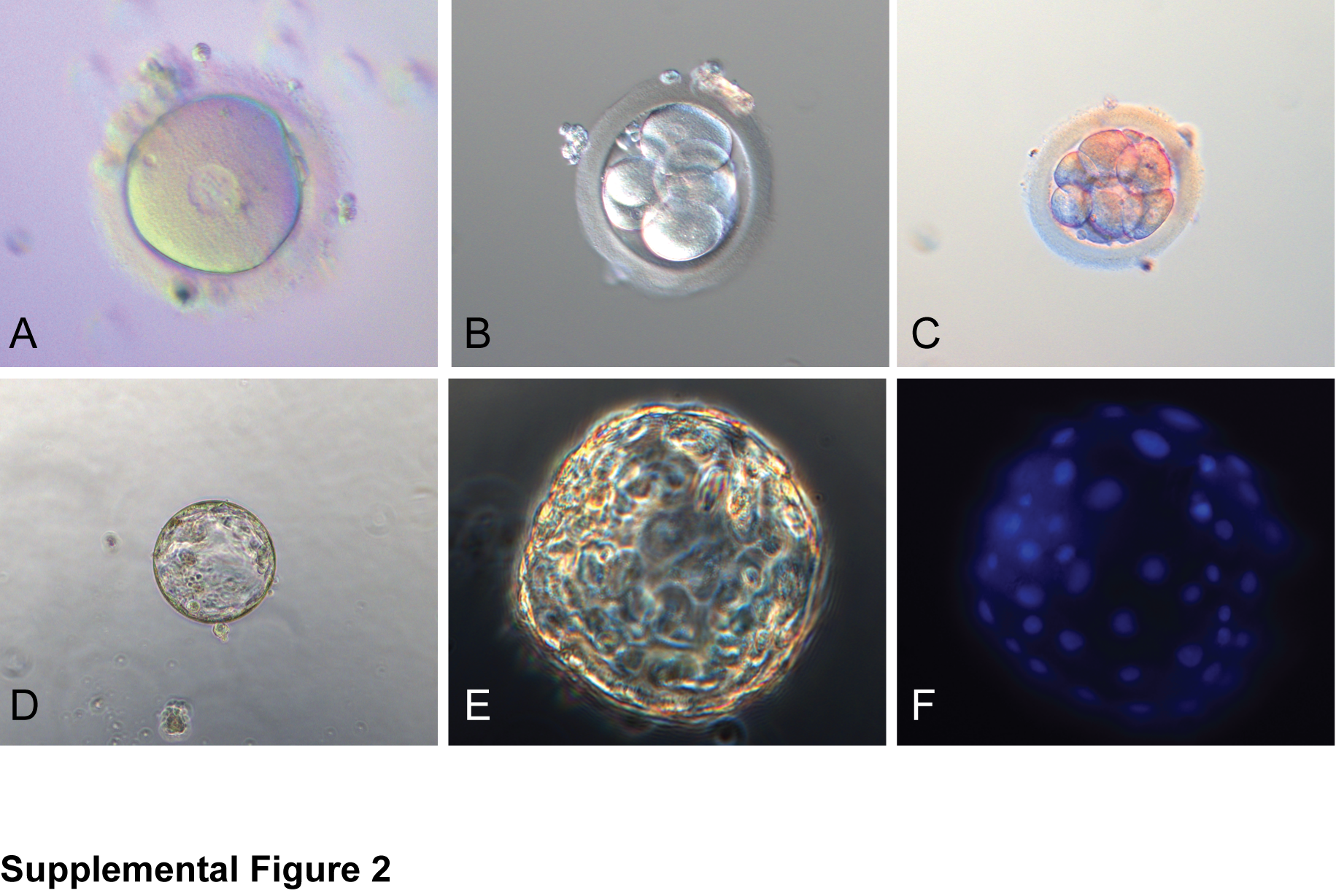

Supplement: Figure S2 — Development of parthenogenic embryos. Embryos on day 1 (A) and day 3 (B,C); blastocysts on day 6 (D) and day 7 (E). Cells were stained with Hoechst 33342 (F). The images were taken either under 300× (A, B, E, F) or 200× (C, D) magnification. (4.97 MB TIF) [file pone.0010979.s002.tif]
