# Supplementary material for: Parthenogenic Blastocysts Derived from Cumulus-Free In Vitro Matured Human Oocytes
Source: PLoS One. 2010 Jun 7;5(6):e10979. doi: 10.1371/journal.pone.0010979 (PMC2881862; doi:10.1371/journal.pone.0010979)
Supplement: Table S1 — Taqman probe assay numbers for genes tested in oocytes and cumulus cells. (0.06 MB DOC) [file pone.0010979.s003.doc]

**Supplemental Table 1. Taqman probe assay numbers for genes tested in oocytes and cumulus cells**

| **Gene symbol** | **Marker** | **Assay ID** |
| --- | --- | --- |
| *NLRP5* | Oocyte specific | Hs00411266_m1 |
| *GDF9* | Oocyte specific | Hs00193364_m1 |
| *ZP1* | Oocyte specific | Hs01399328_m1 |
| *CD14* | Cumulus cell specific | Hs00169122_g1 |
| *FSHR* | Cumulus cell specific | Hs00174865_m1 |
| *LHR* | Cumulus cell specific | Hs00174885_m1 |
| *BDNF* | Ligand | Hs00538277_m1 |
| *IGF-I* | Ligand | Hs01547656_m1 |
| *GDNF* | Ligand | Hs01931883_s1 |
| *Leptin* (*LEP*) | Ligand | Hs00174877_m1 |
| *FGF2* | Ligand | Hs00266645_m1 |
| *GM-CSF* | Ligand | Hs00929873_m1 |
| *EGF* | Ligand | Hs01099999_m1 |
| *TGF-a* | Ligand | Hs00177401_m1 |
| *TGF-b1* | Ligand | Hs00998129_m1 |
| *TGF-b2* | Ligand | Hs01548875_m1 |
| *TGF-b3* | Ligand | Hs00234245_m1 |
| *ET-1* | Ligand | Hs00174961_m1 |
| *ET-2* | Ligand | Hs01012714_m1 |
| *NTRK2* | Receptor | Hs01093103_m1 |
| *NGFRAP1* | Receptor | Hs00276273_s1 |
| *IGF1R* | Receptor | Hs00951562_m1 |
| *GDNFRa* | Receptor | Hs00237133_m1 |
| *RET* | Receptor | Hs01120030_m1 |
| *Leptin recptor* (*LEPR*) | Receptor | Hs00174497_m1 |
| *FGFR2* | Receptor | Hs00240792_m1 |
| *FGFR3* | Receptor | Hs00997393_g1 |
| *FGFR4* | Receptor | Hs00608744_g1 |
| *ESR1* | Receptor | Hs00174860_m1 |
| *ESR2* | Receptor | Hs00230957_m1 |

**Supplemental Table 1. Continue**

| **Gene symbol** | **Marker** | **Assay ID** |
| --- | --- | --- |
| *CSF2Ra* | Receptor | Hs00538900_m1 |
| *CSF2Rb* | Receptor | Hs00166144_m1 |
| *CSF3R* | Receptor | Hs01114427_m1 |
| *EGFR* | Receptor | Hs01076088_m1 |
| *TGF-bR1* | Receptor | Hs00610318_m1 |
| *TGF-bR2* | Receptor | Hs00559660_m1 |
| *TGF-bR3* | Receptor | Hs01114253_m1 |
| *EDNRA* | Receptor | Hs00609865_m1 |
| *GAPDH* | Housekeeping | Hs99999905_m1 |
| *RPLPO* | Housekeeping | 4333761F |
