# Supplementary material for: Parthenogenic Blastocysts Derived from Cumulus-Free In Vitro Matured Human Oocytes
Source: PLoS One. 2010 Jun 7;5(6):e10979. doi: 10.1371/journal.pone.0010979 (PMC2881862; doi:10.1371/journal.pone.0010979)
Supplement: Table S2 — Taqman probe assay numbers for genes tested in embryos. (0.09 MB DOC) [file pone.0010979.s004.doc]

**Supplemental Table 2. Taqman probe assay numbers for genes tested in embryos**

| **Gene symbol** | **Marker** | **Assay ID** |
| --- | --- | --- |
| *MKLP2* | Cytokinesis | Hs00194882_m1 |
| *ECT2* | Cytokinesis | Hs00216455_m1 |
| *COFILIN* | Cytokinesis | Hs00830568_g1 |
| *MYLC2* | Cytokinesis | Hs00853081_g1 |
| *DIAPH1* | Cytokinesis | Hs00946556_m1 |
| *DNMT3A* | DNA methylation | Hs00173377_ml |
| *DNMT2* | DNA methylation | Hs00189402_ml |
| *DNMT3A2* | DNA methylation | Hs00601097_ml |
| *DNMT1* | DNA methylation | Hs00945899_ml |
| *DNMT3L* | DNA methylation | Hs00203536_m1 |
| *DNMT3B* | DNA methylation | Hs00171876_m1 |
| *PRMT5* | Histone modification | Hs00272020_m1 |
| *MSK1* | Histone modification | Hs00178054_m1 |
| *MSK2* | Histone modification | Hs00177670_m1 |
| *AURKB* | Histone modification | Hs00177782_m1 |
| *SETD7* | Histone modification | Hs00363902_m1 |
| *SETDB1* | Histone modification | Hs01051024_g1 |
| *EHMT2* | Histone modification | Hs00198710_m1 |
| *ELP3* | Histone modification | Hs00216429_m1 |
| *KAT5* | Histone modification | Hs00197310_m1 |
| *ATF2* | Histone modification | Hs00153179_m1 |
| *HAT1* | Histone modification | Hs00186320_m1 |
| *GAPDH* | Housekeeping | 4352934E |
| *CTNNB1* | Housekeeping | Hs00170025_m1 |
| *UBE3A* | Imprinting | Hs00166580_m1 |

**Supplemental Table 2. Continue**

| **Gene symbol** | **Marker** | **Assay ID** |
| --- | --- | --- |
| *GNAS* | Imprinting | Hs00255603_m1 |
| *MEG3* | Imprinting | Hs00292028_m1 |
| *H19* | Imprinting | Hs00262142_g1 |
| *IGF2* | Imprinting | Hs00171254_m1 |
| *NDN* | Imprinting | Hs00267349_s1 |
| *PEG1/MEST* | Imprinting | Hs00853380_g1 |
| *SNRPN* | Imprinting | Hs00256087_m1 |
| *PDCD5* | Maternal effect | Hs00270435_m1 |
| *COBL* | Maternal effect | Hs00323596_m1 |
| *NLRP5* | Maternal effect | Hs00411266_m1 |
| *ZAR1* | Maternal effect | Hs00601843_m1 |
| *RNASEN/drosha* | miRNA biogenesis | Hs00203008_m1 |
| *DICER* | miRNA biogenesis | Hs00229023_m1 |
| *DGCR8* | miRNA biogenesis | Hs00256062_m1 |
| *TARBP2* | miRNA biogenesis | Hs00366328_m1 |
| *TERT* | Pluripotency | Hs00162669_m1 |
| *OCT4* | Pluripotency | Hs03005111_g1 |
| *YY1* | Pluripotency | Hs00231533_m1 |
| *CCNA1* | Zygotic activation | Hs00171105_m1 |
| *H2AFZ* | Zygotic activation | Hs01888362_g1 |
| *EIF1AX* | Zygotic activation | Hs00796778_s1 |
| *JARID1B* | Zygotic activation | Hs00366783_m1 |
| *TACC3/maskin* | RNA pathway | Hs00170751_m1 |
| *Symplekin* | RNA pathway | Hs00191361_m1 |
| *CPEB1* | RNA pathway | Hs00229015_m1 |
| *YBX2/Contrin* | RNA pathway | Hs00560265_g1 |
| *AuroraA* | RNA pathway | Hs01582072_m1 |

**Supplemental Table 2. Continue**

| **Gene symbol** | **Marker** | **Assay ID** |
| --- | --- | --- |
| *LEPR* | Growth factors | Hs00174497_m1 |
| *ESR1* | Growth factors | Hs00174860_m1 |
| *NTRK1* | Growth factors | Hs00176787_m1 |
| *ESR2* | Growth factors | Hs00230957_m1 |
| *GDNFR* | Growth factors | Hs00237133_m1 |
| *NGFRAP1* | Growth factors | Hs00276273_s1 |
| *CSF2RA* | Growth factors | Hs00538900_m1 |
| *IGF1R* | Growth factors | Hs00951562_m1 |
| *EGFR* | Growth factors | Hs01076088_m1 |
| *NTRK2* | Growth factors | Hs01093103_m1 |
| *CSF2RB* | Growth factors | Hs00166144_m1 |
| *NTRK3* | Growth factors | Hs00176797_m1 |
| *IGF2R* | Growth factors | Hs00181419_m1 |
| *FGFR2* | Growth factors | Hs00240792_m1 |
| *FGFR1* | Growth factors | Hs00241111_m1 |
| *FGFR4* | Growth factors | Hs00608744_g1 |
| *Insulin receptor* | Growth factors | Hs00961550_m1 |
| *FGFR3* | Growth factors | Hs00997393_g1 |
| *CSF3R* | Growth factors | Hs01114427_m1 |
| *RET* | Growth factors | Hs01120030_m1 |
| *DAZL* | Germ cells | Hs00154706_m1 |
| *PRDM14* | Germ cells | Hs00225842_m1 |
| *DDX4/VASA* | Germ cells | Hs00251859_m1 |
| *IFITM1* | Germ cells | Hs00705137_s1 |
